# Patients with relapsed/refractory chronic lymphocytic leukaemia may benefit from inclusion in clinical trials irrespective of the therapy received: a case-control retrospective analsysis

**DOI:** 10.1038/bcj.2015.78

**Published:** 2015-10-02

**Authors:** D Esteban, N Tovar, R Jiménez, R Santacruz, T Baumann, MaC Pastor, A de la Riva, E Carrera, S Chaves, C Royo, A Navarro, S Rodríguez, C Ayuso, G Riu, N Creus, B Gómez, E Giné, A López-Guillermo, J Delgado

**Affiliations:** 1 Department of Haematology, Hospital Clínic, IDIBAPS, Barcelona, Spain; 2 Haematopathology Unit, Hospital Clínic, IDIBAPS, Barcelona, Spain; 3 Department of Radiology, Hospital Clínic, Barcelona, Spain; 4 Department of Pharmacy, Hospital Clínic, Barcelona, Spain

Treatment for patients with chronic lymphocytic leukaemia (CLL) keeps changing at an incredible pace, perhaps more than in any other haematological malignancy. Indeed, the outlook of patients with CLL is improving with the advent of targeted therapies, including second-generation monoclonal antibodies, immune modulators, BTK inhibitors, PI3K inhibitors and BCL2 antagonists.^1^ After decades of randomized trials showing no benefit in overall survival (OS), recent trials have changed this trend and shown a true OS benefit for patients receiving novel agents.^2, 3, 4, 5^ Moreover, retrospective analyses from our and other institutions have found a significant improvement in survival over the years, suggesting that new treatments are truly changing the prognosis of the disease.^6, 7^

Unfortunately, however, clinical trials pose many ethical dilemmas, result in increased costs over standard care, and not necessarily change clinical practice.^8, 9, 10, 11, 12^ Moreover, experimental therapies may be available outside clinical trials, explaining in part why only 3% of US cancer patients are actually recruited into clinical trials. Indeed, even though it is clear that clinical trials benefit future patients with the disease, there is little evidence to suggest that they benefit those patients that are taking part in them.^13^

The aim of this study was to evaluate the outcome of all consecutive patients with CLL who received second-, third- or fourth-line of therapy at our institution from 2000 to 2014. Therapeutic regimens were divided into those administered in the context of clinical trials and those given outside trials, either because no trial was available at the time or because the patient refused to participate. From our database we identified all consecutive patients with relapsed/refractory CLL who received second-, third- and fourth-line therapy from 2000 to 2014. Patients recruited into prospective clinical trials were considered as cases and the remaining patients were considered as controls. Patients were excluded as controls if at the time of treatment initiation met one or more exclusion criteria that would have made them ineligible for a clinical trial. Specifically, controls were excluded in case of: ECOG performance status greater than 2, severe co-morbidities as defined by one or more organ/system cumulative illness rating scale score of 4, positive hepatitis-B serology, positive hepatitis-C serology, positive HIV serology, prior history of Richter's transformation, prior history of second malignancies (except non-melanoma skin cancer or cervical intraepithelial neoplasia) unless the patient had been disease-free for >5 years, prior history of allogeneic hematopoietic cell transplantation (HCT), and prior history of autologous HCT in the last 6 months. Patients were also excluded as controls if, for any reason not listed above, the therapeutic intention was palliative as documented in the medical notes. Importantly, if a patient received therapy for relapsed/refractory CLL in two or three occasions, he/she was included in the study every time he/she was considered a candidate for a clinical trial. All patients signed a written informed consent approved by the institution's review board. The methods used for the assessment of prognostic factors are detailed in the Supplementary Information.

The distribution of clinical and biological parameters among groups was compared using the Fisher's exact or *χ*^2^ tests. Response to therapy was evaluated using NCI or IWCLL/NCI criteria. Treatment-free survival (TFS) and OS were calculated from treatment initiation using the Kaplan–Meier method. A detailed explanation of the statistical methods is available in the Appendix.

Table 1 shows the patients' baseline characteristics at the time of each therapy line. Both groups were comparable in terms of all prognostic markers evaluated, although there was a trend towards a lower incidence of 17p deletion (4 vs 14%, *P*=0.074) and unmutated *IGHV* genes (71 vs 83%, *P*=0.069) for cases compared with controls. A detailed description of the front-line therapy given to the 162 patients fulfilling the criteria for inclusion in the study is available in Supplementary Table 1. As some patients were treated for relapsed/refractory CLL more than once, the total number of regimens evaluated was 252 (68 within and 184 outside clinical trials, respectively). Of these 252 regimens, 130 (51%), 80 (32%) and 32 (17%) were given as second-, third- and fourth-line therapy, with no significant differences among cohorts (*P*=0.884; Supplementary Table 2). A detailed enumeration of therapeutic regimens administered is shown in Supplementary Table 3.

Overall, there was a trend towards a higher overall response rate for cases compared with controls (73 vs 60%, *P*=0.070), with a significantly higher complete response rate (35 vs 21%, *P*=0.042). Factors associated with a significantly shortened TFS were unmutated *IGHV* genes (*P*<0.001), *TP53* mutations (*P*=0.021), high ZAP70 expression (*P*<0.001), high CD38 expression (*P*=0.004), and 11q deletion (*P*=0.009). Median TFS was significantly longer after inclusion in a clinical trial (28 vs 17 months, *P*=0.025), but the significance was lost when adjusted according to *IGHV* or *TP53* mutational status (*P*=0.099 and *P*=0.093, respectively). Indeed, a multivariate analysis showed that the only factors independently associated with a prolonged TFS were *IGHV* mutational status (hazard ratio (HR) 3.7, 95% confidence interval (CI): 2.0–6.8, *P*<0.001) and *TP53* mutations (HR 2.6, 95% CI: 1.5–4.5, *P*=0.001). Moreover, factors associated with a significantly longer OS were mutated *IGHV* status (*P*<0.001), unmutated *TP53* gene (*P*=0.013), low ZAP70 expression (*P*=0.001), low CD38 expression (*P*=0.001) and receiving treatment in the context of a clinical trial (*P*=0.004). Multivariate analysis confirmed the favourable effect of receiving treatment within a clinical trial (HR 2.6, 95% CI: 1.1–5.7, *P*=0.022) together with having mutated *IGHV* genes (HR 3.9, 95% CI: 1.6–10.1, *P*=0.004) and a wild-type *TP53* gene (HR 2.9, 95% CI: 1.3–6.2, *P*=0.008; Figure 1).

There is no doubt that clinical trials are improving the outlook of patients with leukaemia and other haematological malignancies. At our institution, the number of patients with CLL recruited into clinical trials has dramatically increased in the last years owing to the increasing number of promising molecules and the creation of a clinical trials unit (CTU) in May 2008. This CTU incorporates dedicated physicians, nurses and data managers, which enable us to meet all the demands imposed by sponsors and regulatory authorities. Our study revealed that treatment given in the context of a clinical trial significantly prolonged the survival of patients compared with standard regimens. This observation is surprising because 31% of these therapeutic regimens were administered within phase III trials and, therefore, some of them were EMA-approved and not experimental (for example, ofatumumab, rituximab, bendamustine). On the other hand, both cases and controls received similar front-line therapies, were well balanced in terms of baseline prognostic factors and, in addition, the multivariate analysis confirmed the prognostic relevance of other well-established markers such as *IGHV* and *TP53* mutations, which gives validity to our results.

Even though we included as controls patients who were or could have been eligible for a clinical trial, and we adjusted our results through a multivariate analysis, we cannot rule out the possibility of a bias for cases compared with controls.^14^ This potential bias is the most important limitation of our study as patients entering trials are different from those who do not, owing to many reasons, and it is impossible to adjust for unknown confounders. On the other hand, the difference in response rate is less likely to be influenced by patient selection compared to other endpoints (for example, treatment-free or OS) and hence this is a good hypothesis-generating study.

There are very few studies like ours. A recent paper evaluated the impact of experimental agents on the outcome of patients with relapsed CLL and 17p deletion. Unfortunately, key prognostic factors such as *IGHV* mutational status or beta_2_-microglobulin concentration were not available. Even though this potential bias could not be ruled out, it was concluded that experimental agents improved patients' outcome.^15^ Furthermore, a similar retrospective analysis, but in a completely different disease (Hodgkin's lymphoma), reached a very similar conclusion.^14^

In conclusion, the outcome of patients with relapsed/refractory CLL recruited into clinical trials at our institution was significantly better than that of patients treated outside clinical trials. These results emphasize the importance of clinical trials not only in making progress in the treatment of CLL but also at individual, patient level. Also, our results confirm that survival of patients with CLL is steadily improving, particularly in younger patients.^6, 7^ Finally, clinical trials have been, and will continue being, a key-element in the roadmap leading to further progress in CLL management that should ultimately lead us to cure the disease.

## Figures and Tables

**Figure 1:**
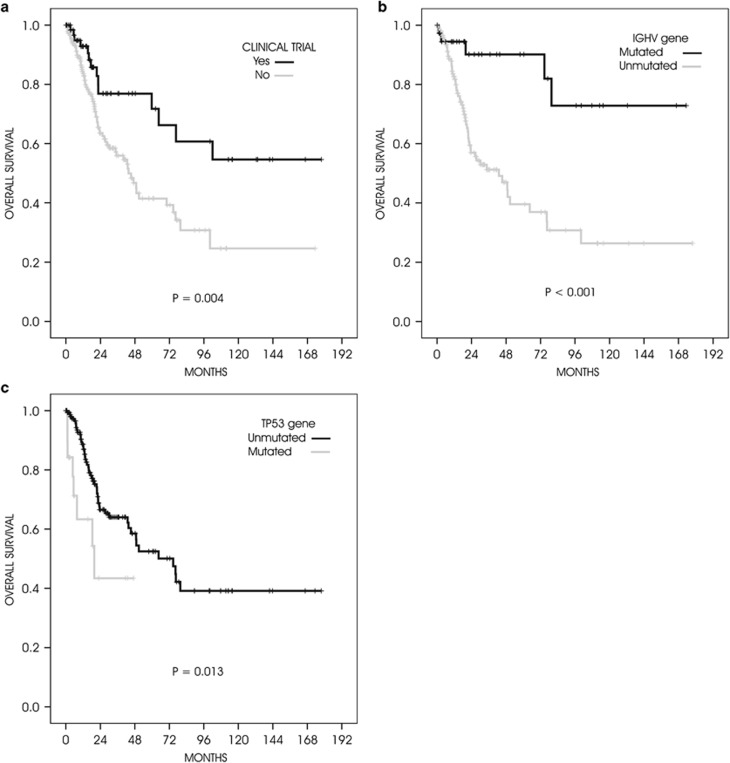
Overall survival. Kaplan–Meier curves of all three factors that had a significant on overall survival by multivariate analysis: (**a**) treatment within a clinical trial (yes vs no); (**b**) *IGHV* gene (mutated vs unmutated); and (**c**) *TP53* gene (mutated vs unmutated).

**Table 1:** Patients' baseline characteristics at the time of each therapeutic regimen

	*Cases (*n=*68)*	*Controls (*n=*184)*	P*-value*
Age (years), median (range)	63 (44–80)	63 (34–88)	0.977
Sex, percentage male/female	59/41	63/37	0.561
Binet stage B/C, *n* (%)	54 (86)	119 (72)	0.088
CIRS score, median (range)	2 (0–8)	3 (0–9)	0.916
Beta_2_-microglobulin, median (range)	2.0 (1.3–6.1)	2.1 (1.0–16.5)	0.374
High ZAP70 expression, *n* (%)	34 (58)	104 (65)	0.350
High CD38 expression, *n* (%)	25 (49)	75 (51)	0.871
Unmutated *IGHV* genes, *n* (%)	37 (71)	115 (83)	0.069
11q deletion by FISH, *n* (%)	9 (17)	38 (24)	0.343
17p deletion by FISH, *n* (%)	2 (4)	22 (14)	0.074
*TP53* mutation, *n* (%)	3 (6)	16 (13)	0.288
*NOTCH1* mutation, *n* (%)	8 (16)	23 (16)	1.0
*SF3B1* mutation, *n* (%)	8 (15)	22 (16)	1.0
Date of therapy initiation, median (range)	September 2004 (January 2000–August 2014)	January 2008 (January 2000–June 2014)	
