# Supplementary material for: Patients with relapsed/refractory chronic lymphocytic leukaemia may benefit from inclusion in clinical trials irrespective of the therapy received: a case-control retrospective analsysis
Source: Blood Cancer J. 2015 Oct 2;5(10):e356–. doi: 10.1038/bcj.2015.78 (PMC4635190; doi:10.1038/bcj.2015.78)
Supplement: Supplementary Information [file bcj201578x1.docx]

**SUPPLEMENTARY INFORMATION**

**Patients with relapsed/refractory chronic lymphocytic leukaemia may benefit from inclusion in clinical trials irrespective of the therapy received: a case-control retrospective analysis**

Daniel Esteban et al.

**Supplemental Methods**

*Prognostic markers*

Fluorescent in-situ hybridization (FISH) studies for 11q, 13q and 17p deletions and gains of chromosome 12 were performed using the Vysis CLL probe kit (Abbott, Des Plaines, IL). Cut-offs used to define alterations were ≥5% for LSI D12Z3 and LSI 13q14.3 probes, and ≥10% for LSI 11q22.3/*ATM* and LSI 17p13.1/*TP53* probes. *IGHV-IGHD-IGHJ* rearrangements and mutational status were analyzed following ERIC recommendations.^1^ For *TP53* mutation analysis we performed direct Sanger sequencing of genomic DNA in four different PCR reactions encompassing exons 4-9.^2^ *NOTCH1* and *SF3B1* mutations were evaluated as previously described.^3,4^ Other clinical and biological characteristics, such as Binet stage, CD38 and ZAP70 expression or beta_2_-microglobulin serum concentration, were evaluated at the time of therapy using conventional methods.

The cumulative illness rating scale (CIRS)^5^ was retrospectively calculated at the time of CLL diagnosis. For this purpose, medical notes were reviewed and each co-morbid condition was graded as recommended by Salvi et al.^6^ Fourteen body systems were evaluated: heart, hypertension, vascular, respiratory, EENT (eye, ear, nose, throat), upper gastrointestinal (GI), lower GI, hepatic, renal, other genital-urinary, muscular-skeletal, neurological, endocrine-metabolic and psychiatric. Each body system was rated from 0 to 4 following these general rules:

1. Absence of disease.
2. Current mild problem or past significant problem.
3. Moderate disability or morbidity requiring first line therapy.
4. Severe problem, significant disability or hard to control chronic problems requiring complex therapeutic regimens.
5. Extremely severe problem, organ failure or severe functional impairment.

The CLL itself was never included in the CIRS, and other malignancies were generally not graded since patients were excluded from this study if they had a second malignancy at the time of therapy.

The CIRS was calculated as the sum of all co-morbid conditions, a number that theoretically ranges from 0 to 56. If a patient had two or more co-morbid conditions in the same body system, only the most severe was graded.

*Statistical analysis*

Treatment-free survival (TFS) was calculated from the date of therapy to the date of next therapy, death or last follow-up. Overall survival (OS) was calculated from the date of therapy to the date of death or last follow-up. Patients undergoing allogeneic hematopoietic cell transplantation were censored the day of the cell infusion.

Curves for TFS and OS were obtained according to the Kaplan-Meier method and the effect of different covariates was evaluated using the log-rank test. These covariates were type of therapy (clinical trial vs. non-clinical trial), *IGHV* mutational status (mutated *vs.* unmutated), *NOTCH1* mutations (presence *vs.* absence), *SF3B1* mutations (presence *vs.* absence), CD38 expression (higher *vs.* lower than 30%), ZAP70 expression (higher *vs.* lower than 20%), FISH aberrations (17p deletion *vs.* 11q deletion *vs.* others), *TP53* mutations (presence *vs*. absence) and B2M (above or below the upper normal range). Cox regression multivariate models were fitted in order to assess the independent prognostic value of those covariates that were significant by univariate analysis. The proportional hazards assumption was visually and statistically checked using Schönfeld residuals.^7^ No multiple imputation methods were used in this analysis. All calculations were performed using SPSS, version 18.0, or R, version 3.0.1. Double sided P values <0.05 were considered significant.

**Supplemental Table 1: Description of frontline treatment regimens administered to all 162 patients included in the study**

|  | **n = 162** |
| --- | --- |
| Fludarabine, cyclophosphamide, mitoxantrone and rituximab | 21 |
| Fludarabine, cyclophosphamide and rituximab | 1 |
| Fludarabine, cyclophosphamide and mitoxantrone | 26 |
| Fludarabine and cyclophosphamide | 4 |
| Fludarabine and rituximab | 1 |
| Fludarabine | 13 |
| Cladribine | 24 |
| Alemtuzumab | 3 |
| Chlorambucil | 48 |
| Chlorambucil and rituximab | 3 |
| Cyclophosphamide, adriamycin, vincristine and prednisone | 9 |
| Cyclophosphamide, adriamycin, vincristine, prednisone and rituximab | 4 |
| Cyclophosphamide, vincritine and prednisone | 1 |
| Bendamustine and rituximab | 1 |
| Dexamethasone | 2 |
| Splenectomy | 1 |

**Supplemental Table 2: Distribution of therapy lines among cases and controls**

|  | **Cases** (n = 68) | **Controls** (n = 184) |
| --- | --- | --- |
| Second line | 36 (53%) | 94 (51%) |
| Third line | 20 (29%) | 60 (33%) |
| Fourth line | 12 (18%) | 30 (16%) |

**Supplemental Table 3: Description of therapeutic regimens**

|  | **Cases**  (n = 68) | **Controls**  (n = 184) |
| --- | --- | --- |
| Fludarabine, cyclophosphamide, mitoxantrone and rituximab | 0 | 25 |
| Fludarabine, cyclophosphamide and rituximab | 0 | 8 |
| Fludarabine, cyclophosphamide, rituximab and lumiliximab | 1 | 0 |
| Fludarabine, cyclophosphamide and ofatumumab | 1 | 0 |
| Fludarabine, cyclophosphamide and mitoxantrone | 37 | 3 |
| Fludarabine and cyclophosphamide | 0 | 6 |
| Fludarabine | 0 | 19 |
| Alemtuzumab | 0 | 24 |
| Ofatumumab | 3 | 0 |
| Rituximab | 0 | 2 |
| Chlorambucil | 0 | 16 |
| Chlorambucil and rituximab | 2 | 4 |
| Chlorambucil and ofatumumab | 1 | 0 |
| Cyclophosphamide, adriamycin, vincristine and prednisone | 0 | 17 |
| Cyclophosphamide, adriamycin, vincristine, prednisone and rituximab | 0 | 20 |
| Cyclophosphamide, vincritine and prednisone | 0 | 3 |
| Cyclophosphamide, vincristine, prednisone and rituximab | 0 | 5 |
| Etoposide, cytarabine, cisplatin, prednisolone and rituximab | 0 | 5 |
| Etoposide, cytarabine, cisplatin and prednisolone | 0 | 4 |
| Dexamethasone, cytarabine, cisplatin and rituximab | 0 | 3 |
| Bendamustine and rituximab | 1 | 18 |
| Bendamustine, rituximab ± idelalisib | 8 | 0 |
| Bendamustine and ofatumumab | 2 | 0 |
| Bendamustine and obinutuzumab | 3 | 0 |
| Ibrutinib | 4 | 0 |
| Lenalidomide | 5 | 0 |
| Dexamethasone | 0 | 2 |

**References**

1. Ghia P, Stamatopoulos K, Belessi C, Moreno C, Stilgenbauer S, Stevenson F, et al. European Research Initiative on CLL. ERIC recommendations on IGHV gene mutational status analysis in chronic lymphocytic leukemia. Leukemia. 2007; 21: 1-3.
2. Pospisilova S, Gonzalez D, Malcikova J, Trbusek M, Rossi D, Kater AP, et al. ERIC recommendations on TP53 mutation analysis in chronic lymphocytic leukemia. Leukemia. 2012; 26: 1458-1461.
3. Puente XS, Pinyol M, Quesada V, Conde L, Ordóñez GR, Villamor N, et al. Whole-genome sequencing identifies recurrent mutations in chronic lymphocytic leukaemia. Nature. 2011; 475: 101-105.
4. Quesada V, Conde L, Villamor N, Ordóñez GR, Jares P, Bassaganyas L, et al. Exome sequencing identifies recurrent mutations of the splicing factor SF3B1 gene in chronic lymphocytic leukemia. Nat Genet. 2011; 44: 47-52.
5. Linn BS, Linn MW, Gurel L. Cumulative illness rating scale. J Am Geriatr Soc 1968; 16: 622–626.
6. Salvi F, Miller MD, Grilli A, Giorgi R, Towers AL, Morichi V, Spazzafumo L, Mancinelli L, Espinosa E, Rappelli A, Dessì-Fulgheri P. A manual of guidelines to score the modified cumulative illness rating scale and its validation in acute hospitalized elderly patients. J Am Geriatr Soc 2008; 56: 1926-1931.
7. Delgado J, Pereira A, Villamor N, López-Guillermo A, Rozman C. Survival analysis in hematologic malignancies: recommendations for clinicians. Haematologica. 2014; 99: 1410-1420.
